# Regarding Tick-Borne Relapsing Fever in the Americas; Some Historical Aspects of a Forgotten Disease in Colombia

**DOI:** 10.3390/vetsci3040033

**Published:** 2016-11-04

**Authors:** Álvaro A. Faccini-Martínez, Carlos A. Botero-García

**Affiliations:** 1 Programa de Pós-Graduação em Doenças Infecciosas, Centro de Ciências da Saúde, Universidade Federal do Espírito Santo, Vitória ES 29000-000, Brazil; 2 School of Medicine, Universidad Militar Nueva Granada, Bogotá 110911, Colombia; bcarlos-10@hotmail.com

**Keywords:** tick-borne relapsing fever, Borrelia, Colombia

## Abstract

In the first decades of the 20th century, scientific papers were published suggesting the presence of Tick-Borne Relapsing Fever in Colombia. As a contribution, we present some historical aspects referring to this topic.

**Dear Editor,**

We have read with great interest the review made by Lopez JE, et al., in which several aspects related to Tick-Borne Relapsing Fever (TBRF) in the Americas are addressed [1]. In this review, despite the presentation of some data regarding South America, existing information was not included in the scientific literature that suggests the presence of TBRF in Colombia. Given the above and as a contribution to this research field, we present some historical aspects referring to this topic.

In Colombia, the first approaches to the local epidemiology of TBRF were published by Franco R, et al. in 1911, suggesting that the tick *Ornithodoros turicata* is the possible vector of the disease in the municipality of Muzo, Department of Boyacá [2]. Subsequently, between 1921 and 1922, based on research conducted in Panamá and Venezuela, researchers suggested the existence of two species of *Borrelia* (*B. neotropicalis* and *B. venezuelensis*) as etiological agents of TBRF in the neotropical region comprised between southern Central America and northern South America, related with *O. talaje* and *O. venezuelensis* as their vectors, respectively [3,4,5]. In addition to this background, in 1923, the entomologist Lawrence H. Dunn, while he was part of a campaign against yellow fever in Colombia, had the opportunity of learning about suspected cases of TBRF diagnosed by Henry Hanson through the visualization of spirochetes in blood smears of patients with febrile illness in the city of Bucaramanga (Department of Santander) [6]. This situation initiated an expedition in different departments of Colombia (Santander, Antioquia, Nariño, Valle del Cauca, Tolima, Cundinamarca, Boyacá, Atlántico and Chocó), completed in July 1924, where a total 4880 specimens of *O. venezuelensis* were captured, of which 2483 (61 pools) were evaluated for the presence and infection by *Borrelia* through the murine model, with 17 pools being positive (27.86%) [6]. During the expedition, Dunn focused his attention upon the observation, in the different towns he visited, of rudimentary constructed houses, which favored the presence in the walls and the parasitism in humans by *O. venezuelensis*, mainly in the departments of the Colombian Pacific region, suggesting an appropriate environment for TBRF in human population, easily confused with Malaria in that geographical region [6].

Interestingly, in 1927, based on his own investigation, and those from other authors, especially in the previous taxonomic description of *O. venezuelensis* by Brumpt in 1921 [7], Dunn published a new manuscript in which he suggested that the ticks previously classified as *O. talaje and O. turicata*, related to the transmission of TBRF in Colombia, Venezuela and Panamá before 1923, actually belonged to the species *O. venezuelensis* [8]. Additionally, in the same manuscript, an approach was made to understand the ecology of *O. venezuelensis* and *O. talaje* (classified at that time as competent vectors for *Borrelia* spp.) in such Latin American countries, describing the former as a common tick in households, whose main host was the human, and the latter, that, despite being present in households (in a lesser extent), mainly fed upon rodents of the genus *Rattus* and rarely humans [8]. Thus, it was recognized that *O. venezuelensis* is the main vector related to the epidemiology of TBRF in northern South America.

On the other hand, as Dunn, Emilio J. Pampana described in the 1920s the clinical context of the TBRF in Colombia after the observation of human cases in the department of Chocó (Colombian Pacific region) [9,10]. Thus, in the period between 1923 and 1926 a total of 91 cases diagnosed through microscopic visualization were documented, of which, 29 patients were foreigners (American or European) and 62 patients were natives; 85% of cases occurred in males and 15% in females. Of the 91 patients, only 38 were followed through time from the onset of symptoms until a month after the last febrile paroxysm (Table 1). The duration of febrile periods in the individuals who were followed was on average of 64 h (36–96 h) in foreign patients and 54 h (24–96 h) in native patients; the first interval between the first and second febrile paroxysms had an average of 10 days (4–27 days). As for the diagnosis by blood smear, the identification of spirochetes was in general scarce. The spirochetes were quickly observable in only 16 patients, mainly in pediatric patients (under 4 years) and with the progression of fever through time. Characteristically, along with the remission of fever, spirochetes disappeared in peripheral blood. Regarding the findings in the blood count, leukocytosis was typically observed in no greater than 12,800 cells during the onset of fever, with subsequent decrease, and leukopenia in the remission of fever. Such leukocytosis was often accompanied by neutrophilia, which also decreased with the lowering number of leukocytes. Leukopenia coincided with increased mononuclear cells and lymphocytes [10]. Symptoms classified as unusual were described, such as seizures, rash and abdominal pain suggestive of appendicitis [9]. There were no related deaths. Pampana highlighted the use of the drug Neosalvarsan as effective in the treatment of disease as it prevented new febrile paroxysms as well as shortening their duration [10].

Years later, with the research work of Osorno-Mesa in 1940, *O. rudis* Karsch, 1880 (previously *O. venezuelensis*) [11] was considered as the vector related to the TBRF with greater geographical distribution in Colombia, with catch records in at least 15 departments, compared to *O. talaje*, the distribution of which remained unclear [12]. In relation to the above, according to data from the World Health Organization in 1989, *O. rudis* was also recognized as a vector of TBRF in the south of Central America and in Venezuela in the border region with Colombia [13], and *B. venezuelensis* was the probable etiologic agent [7].

With this review, we have presented the, even now very little, scientific evidence that suggests the presence of TBRF in Colombia. This could be the initial point for starting new investigations, hand-in-hand with tools such as molecular biology, in order to determine the current distribution of ticks of the genus *Ornithodoros* in Central and South America, the related Borrelias that cause TBRF and possible underdiagnosed human cases or confusion with other causes of acute febrile syndrome [14], as was suggested by Dunn in relation to Malaria in the Colombian Pacific region [6].

## Figures and Tables

**Table 1:** Clinical features of 38 patients diagnosed with Tick-Borne Relapsing Fever (TBRF) in the department of Chocó, Colombia, 1923–1926 [10].

Clinical Features	%
Number of febrile paroxysms	
1	55.3%
2	18.4%
3	23.7%
4	2.6%
Characteristics of the febrile paroxysms **^1^**	
Remittent	46%
Continuous or subcontinuous	27%
Intermittent	27%
Symptoms	
Headache **^2^**	95%
Sweating **^3^**	91%
Chills **^4^**	80%
Osteoarticular pain **^5^**	70%
Myalgia **^6^**	ND
Jaundice **^7^**	70%
Vomiting	70%
Abdominal and Low Back pain	16%
Signs	
Pulse-temperature dissociation (Faget sign)	95%
Bloated face and conjunctival injection	50%
Splenomegaly and hepatomegaly	33%
Pulse dicrotism **^8^**	25%

ND, No data. **^1^** Fever usually ended overnight associated with profuse sweating. **^2^** Severe frontal or retro-ocular pain starting with the onset of fever. **^3^** It often repeated with the remission of fever. **^4^** Often associated with the rise of temperature. **^5^** Early symptom during the course of the disease. **^6^** Pain mainly in the tibial and pretibial region upon palpation. **^7^** Scleral icterus. **^8^** Compared with cases of Malaria that sign was not observed.
